# Insecticidal Activity of Lemongrass Essential Oil as an Eco-Friendly Agent against the Black Cutworm *Agrotis ipsilon* (Lepidoptera: Noctuidae)

**DOI:** 10.3390/insects12080737

**Published:** 2021-08-17

**Authors:** Moataz A. M. Moustafa, Mona Awad, Alia Amer, Nancy N. Hassan, El-Desoky S. Ibrahim, Hayssam M. Ali, Mohammad Akrami, Mohamed Z. M. Salem

**Affiliations:** 1 Department of Economic Entomology and Pesticides, Faculty of Agriculture, Cairo University, Giza 12613, Egypt; moataz.moustafa79@gmail.com (M.A.M.M.); mona.awad2003@gmail.com (M.A.); whitehorse3050@gmail.com (N.N.H.); moat_mon@yahoo.com (E.-D.S.I.); 2 Medicinal and Aromatic Plants Department, Horticulture Research Institute, Agricultural Research Center, Giza 12556, Egypt; dr_aliaamer@yahoo.com; 3 Botany and Microbiology Department, College of Science, King Saud University, P.O. Box 2455, Riyadh 11451, Saudi Arabia; hayhassan@ksu.edu.sa; 4 Department of Engineering, University of Exeter, Exeter EX4 4QF, UK; m.akrami@exeter.ac.uk; 5 Forestry and Wood Technology Department, Faculty of Agriculture (El-Shatby), Alexandria University, Alexandria 21545, Egypt

**Keywords:** *Agrotis ipsilon*, essential oil, enzyme activity, lemongrass, toxicity

## Abstract

**Simple Summary:**

The insect pest, black cutworm, *Agrotis ipsilon* (Lepidoptera: Noctuidae), attacks the seedling stage of many field crops in several countries around the world. To control this insect pest, in this study, lemongrass (*Cymbopogon citratus*) essential oil observed potential insecticidal activity against the second-instar larvae of *A. ipsilon*.

**Abstract:**

Background: The destructive insect pest *Agrotis ipsilon* (Hufnagel) (Lepidoptera: Noctuidae) is a polyphagous species targeting many economically important plants. The extensive and arbitrary use of insecticides has resulted in the build-up of insecticide resistance and pesticide residues accumulating in food. Therefore, it is becoming evident that alternative pest management tools are needed to reduce risks to humans, the environment, and non-target organisms, and at the same time, they should be used in field application at the lowest cost. Methods: In view of this objective, the present study demonstrates the toxicity of lemongrass (*Cymbopogon citratus* (DC.) Stapf) essential oil (EO), against the black cutworm *A. ipsilon* under controlled laboratory conditions in terms of measuring the activity of peroxidase and detoxification enzymes. The chemical components of the EO were analyzed using GC–MS. Results: The results show that after 96 h post treatment, the LC_15_ and LC_50_ values were 427.67 and 2623.06 mg/L, respectively, of *C. citratus* EO on second-instar larvae of *A. ipsilon*. A slight significance in elongation of the larval duration with LC_15_ and LC_50_ value was found with control. By GC–MS analysis, the main compounds identified in the EO were *α*-citral and *β*-citral with percentages of 35.91%, and 35%, respectively. The oxidative stress indicates a significant increase in CAT and lipid peroxidase enzyme activity after 96 h post treatment at the LC_15_ and LC_50_. Conversely, the detoxification enzyme activity shows an inhibition of CarE and GST enzymes of larvae exposed to LC_15_ and LC_50_ values in response to *C. citratus* EO. Conclusions: The present data show that lemongrass EO has insecticidal activity against the black cutworm, *A. ipsilon*.

## 1. Introduction

By 2050, the world population could reach 10 billion [1]. As a result, agricultural producers and researchers are fighting to minimize crop production losses due to insect assault. Currently, management of pest infestations that affect crop yield is often achieved using synthetic chemical insecticides. These chemicals have a negative effect on the agriculture ecosystem, human health [2], and on the prevalence of environmental pollution [3]. Moreover, extensive application of synthetic insecticides led to the destruction of beneficial species such as predators and parasites of the pests, alongside the destruction of honeybees as pollinators, which caused biological imbalance [4,5]. This struggle has resulted in increased research into new crop protection strategies that are cost effective, environmentally friendly, and capable of insect pest management [6]. One of these strategies includes new types of insecticide, such as plant-derived semi chemicals [3], and botanical insecticides [1,6,7], which do not interfere with ecosystems [8], instead of the currently used synthetic insecticides for pest control [9].

Botanical insecticides comprise only 1% of the pesticides market [10], which must be developed. The numbers of plant species that produce chemicals used against pests reached 866 species in 1997 [11]. These chemicals are secondary metabolites that could interfere with some physiological processes and the reproductive system in insect pests [6,12].

Essential oils (EOs) are one of the compounds which are produced as secondary metabolites [13] by aromatic plants and are used in the management of various insect pests and mites [14,15]. Essential oils from the lemongrass family (*Cymbopogon* spp.) are one of about 400–500 commercially produced EOs [16]. The insecticidal property of lemongrass EO is accredited to the various secondary metabolites, such as bioactive cyclic and acyclic terpenes [17], which disrupt the neurotransmitter in insects [18]. Furthermore, other secondary metabolites, such as alkaloids, flavonoids, and carotenoids [19], have been found in lemongrass extracts, indicating its potential as a bio-insecticide. In addition, tannin compounds maybe used as inhibitors of the enzyme activities in insect digestion [20]. Citral (geranial and neral mixture) is considered for the insecticidal activity of lemongrass EO [17,21], resulting from its interaction with oxidative stress and intracellular oxygen radicals [22,23].

The Noctuidae family is the most varied group within Lepidoptera and includes the highest number of species in the agricultural ecosystem [24]. The black cutworm, *Agrotis ipsilon*, is a major, undesirable insect pest, which attacks different field crops, especially at the seedling stage [25], not only in Egypt but also in several other countries. Therefore, to control its populations, an integrated pest management plan is required for efficient and cost-effective management of *A. ipsilon*. Most of the bio or chemical insecticides’ toxicities were evaluated on the development and physiological response in insect pests [26].

Induction of detoxification enzymes such as CytochromP450-dependent mono-oxygenases, esterases, and glutathione-S-transferases, which play key roles in pesticide metabolism, is one of the defensive physiological responses [27]. The induction or inhibition of detoxification enzymes following the application of insecticide could lead to insecticide resistance by raising the insecticide metabolism or secretion rate [28,29]. Additionally, other protective responses, such as oxidative stress enzymes, are responses to chemical stress. These enzymes, such as anti-oxidant and peroxidation enzymes, may stop the deleterious effect of reactive oxygen species (ROS) on cells [30,31].

Therefore, the aim of this study is to verify the existence of a new insecticide against *A. ipsilon*. From this point of view, we studied the lethal and sublethal concentrations of *C. citratus* to evaluate the toxicity, and biological and biochemical activities of lemongrass, *C. citratus,* essential oil on the black cutworm *A. ipsilon* under controlled laboratory conditions for possible use as a safe method of alternative approach to chemical insecticides within an integrated pest control program.

## 2. Materials and Methods

### 2.1. Insect Culture

*A. ipsilon* were reared on castor bean plant for 12 generations in the absence of insecticides in the laboratory at 28 ± 2 °C and 55 ± 5% RH [32,33]. The newly hatched larvae were kept in a clean glass jar (1 L) covered with muslin fixed tightly with a rubber band, and provided daily with castor oil leaves until 3rd-instar larvae. Larvae were reared individually [34] in small plastic cups (7.0 cm in diameter, 3.5 cm in height) with a small piece of castor bean leaf until pupation occurred. The developed pupae were kept in glass jars with paper towels on the bottom and covered until adult emergence.

The newly emerged moths (males and females in ratio 7:5) were transferred to a bigger jar which was supplied with a hanged piece of cotton wool soaked in 10% sugar solution as a dietary supplement [26]. Strips of dark net were used as hanging sites for egg deposition by the mated female moths. The eggs were collected daily and transferred to new jars and left to hatch. The neonates were fed on castor oil leaves and the colony of mass-reared larvae continued as per the above-explained rearing technique. The 2nd-instar larvae of the black cutworm for the initiated treatments were obtained from the colony.

### 2.2. Extraction of C. citratus Essential Oil

Foliage or fresh leaves of *C. citratus*, obtained from the Medicinal and Aromatic Plants Research Department farms, El-Kanater El- Khairiya, Kalubeia Governorate, Egypt, were washed with current tap water to remove the dust then cut to small pieces using excelsior. *C. citratus* EO was hydro-distillated using a Clevenger-type apparatus [35,36,37] from 100 g of fresh sample for 2.5–3.0 h after water heating at 70 °C until no further increase in the EO was observed. A light yellow EO with yield of 2.65% (on a fresh weight basis) was obtained from fresh leaves of lemongrass.

### 2.3. GC–MS Analysis of the Essential Oil

EO composition of the lemongrass was measured with a Trace GC Ultra-ISQ mass spectrometer (Thermo Scientific, Austin, TX, USA) with a direct capillary column TG–5MS (30 m × 0.25 mm × 0.25 µm film thickness). The EO was diluted in *n*-hexane solvent (3 n-heaxane: 1 EO) before being injected to the GC–MS. The used carrier gas was He (flow rate of 1 mL/min). The solvent delay was 3 min, and diluted sample (2 µL) was injected automatically in splitless mode with Autosampler AS1310 coupled with GC. The column oven temperature program and the separation conditions were as follows: At the temperature of 50 °C, the column oven was initially held, then by 5 °C/min, the temperature was increased to 250 °C and held for 2 min. By 25 °C/min, the final temperature was increased to 310 °C and held for 2 min. The temperatures of the injector and MS transfer line were kept at 270 and 260 °C, respectively. At 70 eV ionization voltages, the EI mass spectra were collected at the m/z range of 50–650 in full-scan mode. The temperature of ion source was set at 250 °C.

The Xcalibur 3.0 data system (Thermo Fisher Scientific Inc., Austin, TX, USA, 2014) with the Standard Index and Reverse Standard Index measurements to extract the Match factor from the GC–MS literature is a very intelligent tool to identify chemical constituents, where the value ≥650 is acceptable to confirm the compounds [36,38,39,40,41,42].

### 2.4. Bioassay

*C. citratus* EO was used to treat 2nd-instar larvae of *A. ipsilon*. The stock solution of the EO was prepared in tap water, and a few drops of Tween-20 (10 ppm) was used as an emulsifier. Five different concentrations from the stock of essential oil (8000, 4000, 2000, 1000, and 500 mg/L) were used in the experiment, and water containing the same concentration of Tween 20 was used for the control experiment. Leaves of castor bean were firstly dipped in each of the prepared concentrations for 20 s then left to air dry [43]. Five replicates were used; each one had 10 larvae. The larvae were left to feed for 24 h on the treated leaf, and then, after feeding, the surviving larvae were kept in a clean jar and provided with fresh untreated castor bean leaves. Daily records were taken for larval mortality in order to obtain the lethal and sublethal values after 4 days post treatment.

### 2.5. Lethal and Sublethal Effects

Concentrations of LC_15_ and LC_50_ values of lemongrass, *C. citratus,* were used to determine their effects on developmental stages and adult emergence [43] by using a leaf-dipping technique as described above. After one-week post treatment, the surviving larvae were kept separately in a small clean cup to prevent cannibalism habits, provided with fresh untreated castor bean leaves and covered with muslin (90 larvae for each concentration were used in three replicates) to record the rate of development and growth, including larval and pupal duration, pupation percentage, pupal mortality, pupal weight (three days after pupation), and emergence percentage of the adult [44].

### 2.6. Biochemical Analysis

#### 2.6.1. Oxidative Stress Enzyme Assays

##### Sample Preparation

After 24 and 96 h post treatment of 2nd-instar larvae of *A. ipsilon* by LC_15_ and LC_50_ values of the lemongrass extract oil, 100 mg of fresh body weight of the surviving larvae of *A. ipsilon* were transferred to clean and sterilized Eppendorf tubes (1.5 mL). The samples were stored immediately at −20 °C until later analysis. Each treatment and control sample was replicated five times. The treated larvae were homogenized in potassium phosphate buffer (50 mM, pH 7.0) in a 30 µL buffer per 1 mg body weight. The homogenate was centrifuged for 15 min at 7000× *g* at 4 °C, and the supernatants were used for further analysis.

##### Enzyme Measurement

The catalase (CAT) enzyme activity was estimated [45,46] by measuring the rate of H_2_O_2_ consumption via absorbance at 510 nm. Lipid Peroxidase Assay Kit (Bio-diagnostic Company, Giza, Egypt) was used for monitoring the formation of malondialdehyde (MDA) at 534 nm [47]. The concentration of total protein of all samples was measured spectrophotometrically based on the Biuret Method using a Protein Biuret Kit (Bio-diagnostic Company, Giza, Egypt).

#### 2.6.2. Measurement of Detoxification Enzyme Activity

##### Measurement of Carboxylesterase (CarE) Activity

Activity of CarE (including α- and β- esterase) was determined with some modification [44,48]. Larvae were homogenized in phosphate buffer (40 mM, pH 7.0) and centrifuged at 12,000 *g* at 4 °C for 15 min. A 30 µL aliquot of the supernatant was incubated with 100 µL of (30 mM) α- or β–naphthyl acetate at 25 °C for 15 min. The reaction was stopped by adding 50 µL of stop solution Fast Blue b (2%): sodium dodecyl sulphate (5%). The hydrolysis of *α*-naphthyl acetate was measured at 600 nm, while *β*-naphthyl acetate was measured at 550 nm by Jenway-7205UV/Vis Spectrophotometer. The mean levels of enzyme activity were calculated using Bradford Coomassie Brilliant Blue assay and α- and β-naphthyl acetate standard curves.

##### Measurement of Glutathione S-Transferase (GST) Activity

GST activity was determined with some modification [44,49]. The larvae were homogenized in 0.1 M phosphate buffer (pH 6.5) and centrifuged at 12,000× *g* at 4 °C for 15 min. The solution reaction with 10 µL enzyme stock solution, 25 µL 30 mM CDNB, and 25 µL 50 mM GSH was measured at 340 nm at 25 °C for 3 min using a Jenway-7205 UV/Vis spectrophotometer.

### 2.7. Statistical Analysis

Mortality of the tested larvae was calculated and corrected using Abbott’s formula [50]. The corrected percentages of mortalities were statistically computed according to Probit analysis (EPA Probit analysis program, version 1.5) [51] to estimate the lethal and sublethal values (LC_15_ and LC_50_) of lemongrass on 2^nd^-instar larvae of *A. ipsilon* after four days post treatment. The effects of lemongrass EO on biological and biochemical parameters of *A. ipsilon* were statistically performed using one-way ANOVA with treatment as a fixed effect (the df., F, and *p*-values were established) using SAS software [52]. Mean values of LC_15_ and LC_50_ were analyzed with Duncan’s multiple range test at the 0.05 level of probability and compared with the control.

## 3. Results

### 3.1. Chemical Compound of the Essential Oil from Lemongrass

The chemical compounds of *Cympobogon citratus* EO are presented in Table 1, and Figure S1, where the main compounds were *α*-citral (35.91%), *β*-citral (35.00%), 5-octyldihydro-2(3H)-furanone (9.08%), nerylacetal (7.84%), and *trans*-verbenol (3.58%).

### 3.2. Lemongrass EO Toxicity Test

After 96 h post treatment, the LC_15_ and LC_50_ values were 427.67 and 2623.06 mg/L, respectively (Table 2), of *C. citratus* on 2nd-instar larvae of *A. ipsilon*.

### 3.3. Effects of C. citratus EO on Some Biological Parameters

The results in Table 3 show a slight significance in elongation of the larval duration with LC_15_ and LC_50_ values compared with control. Thus, a significance difference was found in the duration of pupal stage. The pupation % decreased significantly only after the larvae were treated with the LC_50_ value. In contrast, no significant differences were found in the female pupal weight, while the male pupal weight increased at both concentrations. No differences were found in the emergence % and sex ratio (Table 4). It should be noted that sublethal doses/concentrations do not cause insect death, but through the interference in biological traits may reduce the insect populations of the next generations in the crops.

### 3.4. Oxidative Stress Indices

Data in Table 5 indicate that exposure to *C. citratus* caused a significant increase in CAT activity after 96 h post treatment at the LC_15_ and LC_50_ (0.00138 and 0.00151 U/mg of protein, respectively), while the lipid peroxidase enzyme activity at the LC_15_ and LC_50_ were highly significant compared to the control treatment with 0.00192 and 0.00196 mole/mg of protein, respectively. On the contrary, no significance was observed in CAT activity and lipid peroxidase enzyme activity after 24 h from exposure at both values of LC_15_ and LC_50_.

### 3.5. Detoxification Enzymes Activity

CarE and GST dynamic activities were determined after 24 and 96 h post treatment, and the results are shown in Table 6. The activity of CarE after 24 h showed that *α*-esterase has no significant differences compared to the control treatment, while the *β-*esterase showed significant differences after 24 h post treatment at the LC_15_ and LC_50_ (14.59 and 13.45 μmole/min/mg of protein, respectively). After 96 h post treatment, the activity of CarE showed a different pattern of change; the *α*-esterase showed significant differences compared to the control treatment (10.48 and 9.46 μmole/min/mg of protein, respectively) at the LC_15_ and LC_50_, while the *β*-esterase decreased significantly compared to the control treatment at the LC_50_ (8.65 μmole/min/mg of protein). In addition, the GST activity after 24 h showed a significant decrease at the LC_50_ (9.36 μmole/min/mg of protein), while the activity was significantly decreased at the LC_15_ and LC_50_ (8.80 and 7.00 μmole/min/mg of protein, respectively).

## 4. Discussion

The EO chemical analysis of lemongrass (Table 1) revealed a rich citral profile [53]. Citral was found in the leaf EO as majora major component compared to other compounds such as limonene, geraniol, and citronellal [54,55]. Previously, *β*-citral, geranial (*α*-citral or citral A), and *β*-myrcene, with percentages of 43.63, 41.51, and 12.37%, respectively, were identified as the main compounds in EO of *C. citratus* collected from Alexandria, Egypt [56]. Additionally, the plant grown in Giza, Egypt, showed that geranial (40.72%), neral (34.98%), and myrcene (15.69%) were the main compounds from the fresh leaves [57]. Geranial, neral, and myrecene were the main compounds in leaf EO from lemongrass collected from Kenya with percentages of 39.53, 33.31, and 11.41%, respectively [58], and geranial (42.2%), and neral (31.5%) from the Algerian plant [59]. With the same pattern, *C. citratus* leaves from Togo and Brazil showed the presence of geranial (45.2, and 47.52%), and neral (32.4 and 35.54%), respectively [60,61] as main compounds in the EO. Recently, neral, geranyl acetate, geranial, limonene, camphene, and citronellal, at 24.60, 18.70, 12.40, 12.30, 7.55, 4.70, and 3.21%, respectively, were shown to be the main compounds of *C. citratus* EO from Brazil [62]. The EO from Brazilian and Cuban studies showed the presence of *Z*–citral (neral) and *E*–citral (geranial) with percentages of 36.37, 35.21, and 53.2, 51.14%, respectively [63]. Furthermore, neral, citral, nonan-4-ol, camphene, 6-metil-hept-5-en-2-one, citronelal, *β*-caryophyllene, citronelol caryophyllene oxide, *γ*-muurolene, limonene, geranyl acetate, and geranial were identified as the primary compounds of the lemongrass EO with percentages of 31.5, 26.1, 6.54, 5.19, 4.36, 3.83, 3.26, 2.95, 2.63, 2.46, 2.32, 2.27, and 2.15%, respectively [64]. To find alternatives to toxic synthetic insecticides, this study evaluated the lethal and sublethal concentration of lemongrass EO on the survivorship, biological parameters, and enzyme activity of the destructive pest *A. ipsilon*. The leaf-dip bioassay revealed that the LC_15_ and LC_50_ values were 427.67 and 2623.06 mg/L, respectively, of *C. citratus* on the second-instar larvae of *A. ipsilon*. This might be due to the citral compounds, which inhibited acetylcholinesterase and octopamine [65,66,67], causing insect paralysis and death. The toxic effect of lemongrass EO on the life cycle of insects belonging to the orders Hemiptera [68], Coleoptera [3,69], Lepidoptera [70], and Diptera [63] have been reported. In addition, pest life-history components, behavior, and physiology can be significantly exhibited by the sublethal concentrations of EOs [71].

*C. citratus* EO has a toxic effect on *Spodoptera frugiperda*, where the LC_50_ was 1.35 mg/L, while the LC_50_ was 1.042 mg/L after treating the larvae with citral from *C. flexuosus* [72]. Park et al. [73] found that LC_50_ of the EOs of *C. aurantium* fruits was 92.58 and 113.26 mg/L against *Pochazia shantungensis* nymphs and adults, respectively. Additionally, Pumnuan and Insung [74] revealed that the fumigant toxicity of citral against thrips and mealybugs was LC_50_ = 2.31 and 2.80 µL/L air, respectively. From first to fifth instars of *Podisus nigrispinus* (Heteroptera: Pentatomidae) nymphs, the increase in LD_50_ of 1.08 to 139.30 μg/insect^−1^ and LD_90_ of 2.02 to 192.05 μg/insect^−1^ was found as lemongrass EO was applied [64].

A set of results points to the toxicological effects of lemongrass EO on insect pests with LC_50_ of 35.133 mg/L against third-instar larvae of *Aedes aegypti* mosquitoes [75], 0.268 ppm against the adult moth of *Phthorimaea operculella* [76], and 7.07 ppm against the adult stage of cowpea weevil *Callosobruchus maculatus* [13]. Thus, the lemongrass EO has shown toxic effects on adults of *Sitophilus granaries* (LD_50_ = 4.03 µg/insect^−1^) [62]. For the toxicity of lemongrass commercial constituents, geranyl acetate showed lower toxicity than citral in third-instar *P*. *nigrispinus* nymphs, with LD_50_ 33.44 μg/insect^−1^ and LD_90_ 48.34 μg/insect^−1^, compared to LD_50_ 25.56 μg/insect^−1^ and LD_90_ 35.39 μg/insect^−1^ for the citral [64].

Accordingly, this confirmed the significant elongation of the larval and pupal duration for exposed larvae to LC_15_ and LC_50_ values of *C. citratus*. Likewise, EOs such as garlic exhibited anti-feeding and starvation effects against the black cutworm, *A. ipsilon* (Lepidoptera: Noctudae), with LC_50_ ranging from 0.006 to 0.019%; additionally, the combination of the EOs (garlic + mint) exhibited a potentiating effect and the toxicity was increased [77]. Similarly, *Eucalyptus* EO was effective as an anti-feeding deterrent against the third-instar larvae of *A. ipsilon* and *Spodoptera littoralis* [78]. Additionally, EO of garlic, mint, cumin, caraway, and parsley had antifeedant and starvation effects after treating the larvae of *A. ipsilon* with the LC_50_ value [77].

In contrast, no statistically significant difference in the emergence (%) relative to control was recorded in all other treatments. On the contrary, the cowpea weevil, *Callosobruchus maculatus*, showed a significant decrease in the emergence of adults at all concentrations of lemongrass EO tested [3].

The oxidative stress enzyme activity reveals the lack of significant activity observed in CAT and lipid peroxidase at 24 h post treatment in LC_15_ and LC_50_ equivalent. On the contrary, the expression level of CAT and lipid peroxidase after 96 h post treatment showed an increase in the activity of CAT of approximately 1.104- and 1.208-fold, respectively, compared to the control treatment of LC_15_, and LC_50_. This indicates an overproduction of hydrogen peroxide (H_2_O_2_) in the insect’s body [79]. The antioxidant defense plays an important role in combating reactive oxygen species (ROS) and thus protecting the cell from ROS-mediated oxidative attack. In this way, the lipid peroxidase shows the same pattern, with increased activity of about 1.04- and 1.05-fold, respectively, at the LC_15_, and LC_50_.

On the other hand, insects could be adapted to various biochemical and physiological mechanisms to withstand the internal and external stress in order to survive. Generally, detoxifying enzymes are important for the metabolism of xenobiotics in insects [80,81]. Inhibition of the detoxification enzymes CarE and GST in response to *C. citratus* larvae exposed to LC_15_ and LC_50_ values were observed in our study. However, increased levels of both CarE and GST enzymes were observed in arthropod lines that demonstrated resistance to the insecticides [82,83].

## 5. Conclusions

This study demonstrates the potential of lemongrass EO as an insecticide approach to management of *A. ipsilon*. Lemongrass oil caused significant effects on the mortality, developmental duration, and expression level of CAT and lipid peroxidase after 96 h post treatment. Conversely, treated larvae showed inhibition of detoxification enzymes compared with the control larvae. The above findings of the present study show that the EO of lemongrass has a wide spectrum of biological activities, and also produces an enzymatic defense response. Moreover, these biochemical changes could be considered as biochemical indicators for oil-treatment stress. EO of lemongrass showed toxic and sublethal effects on *A. ipsilon*, and can be used as an alternative to synthetic insecticides. However, further investigations are underway to evaluate *C. citratus* under field condition.

## Figures and Tables

**Table 1:** Phytochemical screening of essential oil from lemongrass leaves by GC–MS analysis (Figure S1).

RT	Area%	Compound Name	Match Factor (MF)
7.97	0.83	Isoneral	850
8.37	1.49	Isogeranial	900
8.60	0.71	Dihydronopol	750
9.77	35.00	Neral or *β*-citral (Citral B)	955
10.47	35.91	Geranial or *α-*citral (Citral A)	850
10.61	3.58	*trans*-Verbenol	750
10.71	0.91	Epoxy-linalooloxide	800
10.97	1.45	Geranyl vinyl ether	955
11.26	7.84	Nerylacetal	929
11.76	9.08	5-Octyldihydro-2(3H)-furanone	930
12.69	1.24	Geraniol acetate	950
13.74	1.24	(Z,E)-*α*-farnesene	939
16.77	0.72	*β*-Caryophyllene epoxide	850

RT: Retention time (min).

**Table 2:** Toxicity of *C. citratus* essential oil on the 2nd-instar larvae of *A. ipsilon.*

LC_15_ (mg/L) (95% Confidence Limit)	LC_50_ (mg/L) (95% Confidence Limit)	Slope ± SE	ꭓ^2^
427.67(134.84–746.35)	2623.06 (1797.26–4144.71)	1.31 ± 0.26	0.72

ꭓ^2^: Chi square.

**Table 3:** Effects of *C. citratus* essential oil on the development of larval and pupal stages after treating the 2nd-instar larvae of *Agrotis ipsilon* with LC_15_ and LC_50_ values.

Treatments	Mean ± SE				
	Larval Duration	Pupal Duration	Pupation%	Pupal Weight (g)	
				Female	Male
Control	20.36 ± 0.17 ^a^*	18.24 ± 0.24 ^a^	100 ± 0.0 ^a^	0.36 ± 0.02 ^a^	0.35 ± 0.01 ^b^
LC_15_	21.20 ± 0.18 ^b^	18.70 ± 0.12 ^b^	100 ± 0.0 ^a^	0.41 ± 0.01 ^a^	0.39 ± 0.007 ^a^
LC_50_	21.28 ± 0.17 ^b^	19.57 ± 0.13 ^c^	94.81 ± 1.01 ^b^	0.40 ± 0.01 ^a^	0.40 ± 0.007 ^a^
F	7.33	16.50	26.16	1.84	5.75
*p*-value	0.0008	<0.0001	0.0011	0.165	0.0043

* Means with the same letter within the same column are not significantly different according to Duncan’s multiple range test at 0.05 level of probability. SE: Standard error.

**Table 4:** Effects of *C. citratus* essential oil on the emergence %, and sex ratio after treating the 2nd-instar larvae of *Agrotis ipsilon* with LC_15_ and LC_50_ values.

Treatments	Mean ± SE		
	Emergence%	Sex Ratio	
		Female	Male
Control	98.61 ± 1.39 ^a^*	60.94 ± 7.04 ^a^	39.06 ± 7.04 ^a^
LC_15_	98.48 ± 1.51 ^a^	45.11 ± 5.32 ^a^	54.89 ± 5.32 ^a^
LC_50_	97.09 ± 1.45 ^a^	44.45 ± 5.69 ^a^	55.55 ± 5.69 ^a^
F	0.33	2.37	2.37
*p*-value	0.728	0.174	0.174

* Means with the same letter within the same column are not significantly different according to Duncan’s multiple range test at 0.05 level of probability. SE: Standard error.

**Table 5:** Mean (±SE) of catalase, and lipid peroxidase enzyme activity of *A. ipsilon* after 24 and 96 h of 2nd-instar larvae being exposed to LC_15_ and LC_50_ values of *C. citratus* essential oil.

Treatments	Mean ± SE			
	Catalase (U/mg of Protein)	Lipid Peroxidase (mole/mg of Protein)	Catalase (U/mg of Protein)	Lipid Peroxidase (mole/mg of Protein)
	24 h		96 h	
Control	0.00138 ± 0.00003 ^a^*	0.00181 ± 0.00004 ^a^	0.00125 ± 0.00002 ^b^	0.00185 ± 0.00002 ^b^
LC_15_	0.00151 ± 0.00001 ^a^	0.00196 ± 0.00014 ^a^	0.00138 ± 0.00009 ^ab^	0.00192 ± 0.000005 ^ab^
LC_50_	0.00153 ± 0.00008 ^a^	0.00171 ± 0.00004 ^a^	0.00151 ± 0.00005 ^a^	0.00196 ± 0.00002 ^a^
F	3.08	1.87	3.73	7.33
*p*-value	0.1203	0.2337	0.0886	0.0245

* Means with the same letter within the same column are not significantly different according to Duncan’s multiple range test at 0.05 level of probability.

**Table 6:** Mean (±SE) of detoxification enzymes (carboxylesterase and GST) activity of *A. ipsilon* after 24 and 96 h of 2nd-instar larvae being exposed to LC_15_ and LC_50_ values of *C. citratus* essential oil.

Treatments	Mean ± SE					
	Carboxylesterase (μmole/min/mg of Protein)		GST (μmole/min/mg of Protein	Carboxylesterase (μmole/min/mg of Protein)		GST (μmole/min/mg of Protein
	*α-*Esterase	*β*-Esterase		*α*-Esterase	*β*-Esterase	
	24 h			96 h		
Control	13.24 ± 0.87 ^a*^	17.09 ± 1.26 ^a^	14.09 ± 0.47 ^a^	12.78 ± 0.62 ^a^	14.53 ± 1.16 ^a^	12.46 ± 0.28 ^a^
LC_15_	9.36 ± 0.52 ^b^	14.59 ± 0.86 ^ab^	9.62 ± 0.49 ^b^	10.48 ± 0.55 ^ab^	13.25 ± 1.30 ^a^	8.80 ± 0.65 ^b^
LC_50_	8.79 ± 0.40 ^b^	13.45 ± 0.16 ^b^	9.36 ± 0.91 ^b^	9.46 ± 0.91 ^b^	8.65 ± 0.21 ^b^	7.00 ± 0.78 ^b^
F	14.52	4.37	16.19	5.63	7.37	20.47
*p*-value	0.0050	0.0673	0.0038	0.0420	0.0219	0.0021

* Means with the same letter within the same column are not significantly different according to Duncan’s multiple range test at 0.05 level of probability. SE: Standard error.

## Supplementary Materials

The following are available online at https://www.mdpi.com/2075-4450/12/8/737/s1, Figure S1: title. GC-MS chromatogram of the identified chemical compounds in the essential oil from *Cympobogon citratus* leaves.
