# Correction: Moustafa et al. Insecticidal Activity of Lemongrass Essential Oil as an Eco-Friendly Agent against the Black Cutworm *Agrotis ipsilon* (Lepidoptera: Noctuidae). *Insects* 2021, *12*, 737

**DOI:** 10.3390/insects12110991

**Published:** 2021-11-04

**Authors:** Moataz A. M. Moustafa, Mona Awad, Alia Amer, Nancy N. Hassan, El-Desoky S. Ibrahim, Hayssam M. Ali, Mohammad Akrami, Mohamed Z. M. Salem

**Affiliations:** 1 Department of Economic Entomology and Pesticides, Faculty of Agriculture, Cairo University, Giza 12613, Egypt; moataz.moustafa79@gmail.com (M.A.M.M.); mona.awad2003@gmail.com (M.A.); whitehorse3050@gmail.com (N.N.H.); moat_mon@yahoo.com (E.-D.S.I.); 2 Medicinal and Aromatic Plants Department, Horticulture Research Institute, Agricultural Research Center, Giza 12556, Egypt; dr_aliaamer@yahoo.com; 3 Botany and Microbiology Department, College of Science, King Saud University, P.O. Box 2455, Riyadh 11451, Saudi Arabia; hayhassan@ksu.edu.sa; 4 Department of Engineering, University of Exeter, Exeter EX4 4QF, UK; m.akrami@exeter.ac.uk; 5 Forestry and Wood Technology Department, Faculty of Agriculture (El-Shatby), Alexandria University, Alexandria 21545, Egypt

**Additional Affiliations**

In the published publication [1], there was an error regarding the affiliations for **Hayssam M. Ali**. In the original version of this article, Hayssam M. Ali was incorrectly affiliated with ‘Timber Trees Research Department, Sabahia Horticulture Research Station, Horticulture Research Institute, Agriculture Research Center, Alexandria, Egypt’. This affiliation has be removed. The correct affiliation is listed below.

Botany and Microbiology Department, College of Science, King Saud University, P.O. Box 2455, Riyadh 11451, Saudi Arabia

The authors apologize for any inconvenience caused and state that the scientific conclusions are unaffected. The original publication has also been updated.
