# Supplementary material for: Insecticidal Activity of Lemongrass Essential Oil as an Eco-Friendly Agent against the Black Cutworm Agrotis ipsilon (Lepidoptera: Noctuidae)
Source: Insects. 2021 Aug 17;12(8):737. doi: 10.3390/insects12080737 (PMC8396863; doi:10.3390/insects12080737)
Supplement: Supplementary file 1 [file insects-12-00737-s001.zip › insects-1317220-supplementary.pdf]

Insecticidal Activity of Lemongrass Essential Oil as an Eco-friendly Agent Against the Black Cutworm *Agrotis ipsilon* (Lepidoptera: Noctuidae)

Moataz A. M. Moustafa [1], Mona Awad [1], Alia Amer [2], Nancy N. Hassan [1], El-Desoky S. Ibrahim [1], Hayssam M. Ali [3,4], Mohammad Akrami [5] and Mohamed Z. M. Salem [6,*]

[1] Department of Economic Entomology and Pesticides, Faculty of Agriculture, Cairo University, 12613 Giza, Egypt; moataz.moustafa79@gmail.com (M.M.); mona.awad2003@gmail.com (M.A.); whitehorse3050@gmail.com (N.N.H.); moat_mon@yahoo.com (E.D.S.I.)
[2] Medicinal and Aromatic plants Department, Horticulture Research Institute, Agricultural Research Center, Giza, Egypt (A.A.: dr_aliaamer@yahoo.com)
[3] Botany and Microbiology Department, College of Science, King Saud University, P.O. Box 2455, Riyadh 11451, Saudi Arabia; hayhassan@ksu.edu.sa
[4] Timber Trees Research Department, Sabahia Horticulture Research Station, Horticulture Research Institute, Agriculture Research Center, Alexandria 21526, Egypt
[5] Department of Engineering, University of Exeter, Exeter EX4 4QF, UK; m.akrami@exeter.ac.uk (M.A.)
[6] Forestry and Wood Technology Department, Faculty of Agriculture (El-Shatby), Alexandria University, Alexandria 21545, Egypt; zidan_forest@yahoo.com (M.Z.M.S.)
* Correspondence: zidan_forest@yahoo.com; mohamed-salem@alexu.edu.eg

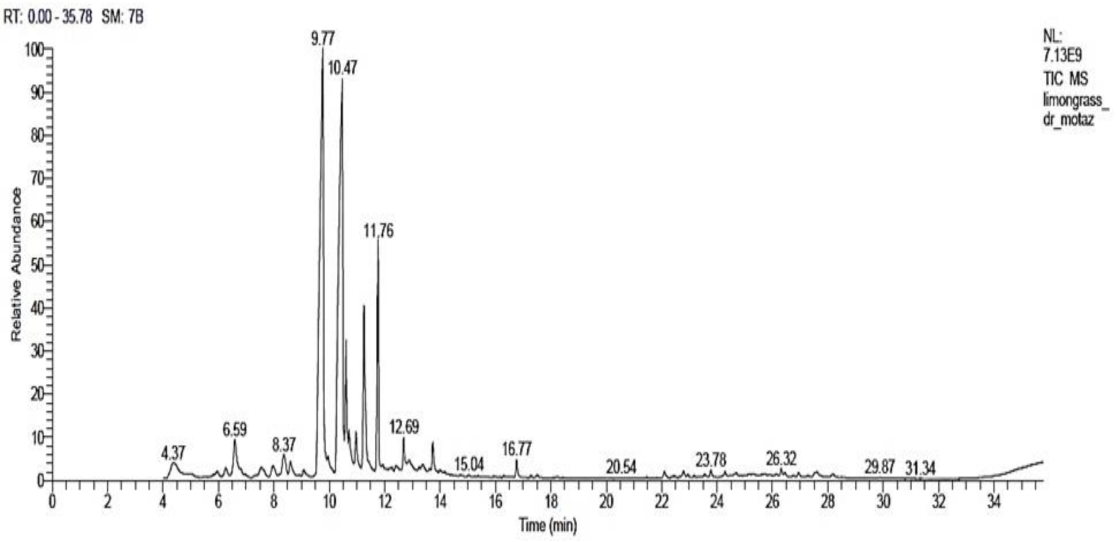

Figure S1. GC-MS chromatogram of the identified chemical compounds in the essential oil from *Cympobogon citratus* leaves.
